# Accuracy of Intraocular Lens Power Calculation Using Anterior Chamber Depth from Two Devices with Barrett Universal II Formula

**DOI:** 10.1155/2019/8172615

**Published:** 2019-09-23

**Authors:** Hannah Muniz Castro, Audrey X. Tai, Samuel J. Sampson, Matthew Wade, Marjan Farid, Sumit Garg

**Affiliations:** ^1^UT Health Science Center, Houston, TX, USA; ^2^UC Irvine Department of Ophthalmology, Gavin Herbert Eye Institute, Irvine, CA, USA; ^3^Kaiser Permanente Los Angeles Medical Center, Los Angeles, CA, USA

## Abstract

**Purpose:**

To compare the preoperative measurements of the anterior chamber depth (ACD) by the IOLMaster and Catalys; additionally, to compare the accuracy of the IOL power calculated by the Barrett Universal II formula using the two different measurements.

**Setting:**

University of California, Irvine, Gavin Herbert Eye Institute in Irvine, California.

**Design:**

Retrospective comparative study.

**Methods:**

This study included 144 eyes of 90 patients with a mean age of 72.0 years (range 40.8 to 92.1 years) that underwent femtosecond laser-assisted cataract surgery using Catalys. Preoperative measurements of ACD were taken by the IOLMaster and Catalys. Manifest refraction and refractive spherical equivalent were measured 1 month postoperatively. Expected refractive results were compared with actual postoperative refractive results.

**Results:**

The correlation between the ACD values from the two devices was good (*r* = 0.80). The Catalys ACD measurements yielded a larger ACD compared to the IOLMaster, with a mean difference of 0.22 mm (*P* < 0.0001). The correlation between the postoperative and predicted RSE of the implanted IOL power was excellent (*r* = 0.96). There was no statistically significant difference between the mean absolute error derived from the IOLMaster, 0.37 diopter (D) ± 0.34 (SD), and the Catalys, 0.37 ± 0.35 D (*P*=0.50).

**Conclusions:**

The Catalys biometry yielded a significantly larger ACD value than the IOLMaster. This difference in ACD value, however, did not reflect in a statistically significant difference in IOL power calculation and refractive prediction error using the Barrett Universal II Formula.

## 1. Introduction

As previous research have noted, postoperative visual acuity (VA), and therefore patient satisfaction in cataract surgery, is largely dependent upon accurate selection of intraocular lens (IOL) power [1, 2]. IOL power selection in turn relies upon the accurate prediction of effective lens position (ELP), which is defined as the distance between the anterior surface of the cornea and the infinitely thin lens plane. Besides being directly dependent on measurable preoperative biometry of anterior chamber depth (ACD), ELP is also impacted by operative and postoperative factors such as surgical technique and postoperative settling of the IOL [3]. These discrete terms are estimated by different methods depending on the predictive IOL power formula used.

Several of these predictive IOL power formulas have been developed in efforts to increase the formulaic accuracy and applicability to a wide range of eye measurements. However, despite advances in biometer technology and IOL formulas, prediction of ELP remains challenging [4, 5]. Errors in predicting the ELP are the main contributors to inaccurate IOL power selection and are estimated at approximately 35–42% of total postoperative refractive error [6, 7]. A 0.25 mm error in postoperative ACD prediction will result in an IOL power error from 0.10–0.55 D, depending on the overall axial length of the eye [7].

For the decade 1999–2009, the IOLMaster (Carl Zeiss Meditec AG), an ophthalmic biometer based on partial coherence interferometry (PCI), was the only device of its kind on the market utilizing optical technology for ophthalmic biometry [8]. The IOLMaster measures the ACD using a nonlaser optical lateral slit illumination [9]. After its introduction in 1999, multiple studies documented the high reliability of its biometric measurements, making it the biometry gold standard for many years [2, 10–12].

Since that time, many other optical biometers have been released utilizing various optical technologies [8, 13]. Introduced in 2012, the Catalys system (Johnson & Johnson Surgical Vision Inc., Santa Ana, CA) is a relatively new device that integrates a femtosecond laser capsulotomy/phacoemulsification system with proprietary long-range spectral-domain optical coherence tomography (OCT) and near-infrared video imaging. A three-dimensional map of the lens and anterior chamber is acquired with the OCT, allowing the determination of intraoperative biometric measurements prior to laser delivery [11, 13].

As alluded to above, the choice of IOL power formula is also a critically important factor in achieving accurate postoperative refractive outcomes, as each formula determines ELP utilizing different theoretical models. Several are used in clinical practice today [10]. The Barrett Universal II is a fourth-generation formula that uses five inputs in the calculation algorithm: ACD, axial length (AL), keratometry (K) values, lens thickness, and white-to-white distance (WTW) [14]. Recent studies have demonstrated more accurate prediction outcomes using the Barrett Universal II compared to other IOL power formulas [15, 16].

We designed this retrospective study to assess for any significant differences in ACD measurements between the IOLMaster and Catalys OCT system in patients undergoing femtosecond laser-assisted cataract surgery. Additionally, we compared the accuracy of IOL power calculation using the ACD measurements from both devices utilizing the Barrett Universal II formula.

## 2. Materials and Methods

A retrospective chart review of all patients who underwent femtosecond laser-assisted cataract surgery at an academic institution between August 2015 and July 2016 was performed. Institutional Review Board approval was obtained before the initiation of the study.

To determine the minimal sample size for statistically significant results, power analysis was performed. Assuming a standard deviation of 0.40 D and performing a 2-tailed test, an estimated sample size of 94 was needed to achieve an alpha level of 0.05 and power of 80%.

Inclusion criteria were (1) preoperative IOLMaster 500 biometric measurements; (2) preoperative Catalys biometric measurements; (3) uneventful cataract surgery; and (4) postoperative manifest refraction at least 1 month after surgery. Exclusion criteria were (1) perioperative complications and (2) incomplete biometric or refractive data.

All participants were assessed at least 1 month postoperatively by qualified technicians. Manifest refraction was obtained and the postoperative refractive sphere equivalent (RSE) was calculated for each patient. The mean ACD was calculated and compared between devices.

To evaluate predictability between biometers, the expected refractive results were compared with the actual postoperative refractive results. The predicted RSE was given by the Barrett Universal II formula, which was accessed through the online calculator at http://www.apacrs.org/barrett_universal2 [14]. The prediction error in IOL power calculation was measured by subtracting postoperative RSE from the predicted RSE of the implanted IOL, taking the ACD measured from each device into account. The mean absolute error (MAE) was calculated for each device and defined as the average absolute value of the prediction error. The MAEs were compared to assess for predictive accuracy. The percentage of eyes within 0.25 D, 0.5 D, 0.75 D, and 1.0 D were calculated for each device. Bland–Altman plots were used to determine the agreement between devices. Fisher's exact test was used to determine whether the proportion of eyes achieving an RSE less than 0.5 D differed between the Catalys and IOLMaster. Statistical significance was defined as a *P* value less than 0.05 (2-tailed).

## 3. Results

**Figure 1:**
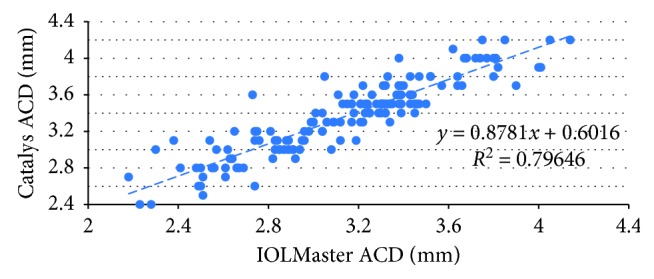
Correlation between ACD measurements taken from both devices.

**Figure 2:**
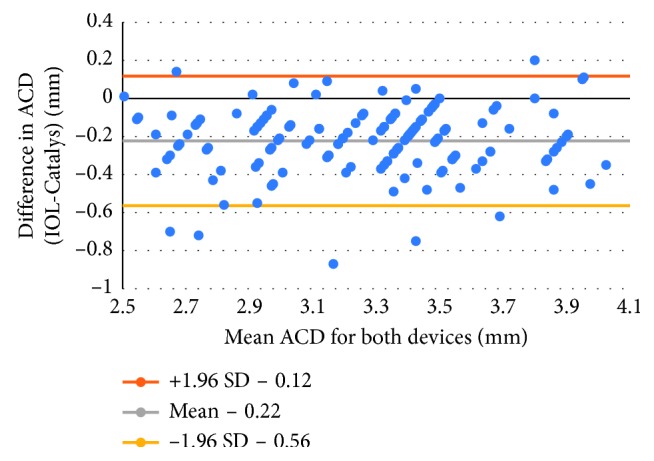
Bland–Altman plot of ACD values for both devices.

This case series included 144 eyes of 90 patients, of which 66 were female. The mean age was 72.0 years (range 40.8 to 92.1 years). In total, 71 left eyes and 73 right eyes were measured.  Table 1 shows the mean, standard deviation, minimum and maximum ACD values of each device and their difference. The Catalys measurements yielded a larger ACD compared to the IOLMaster, with a mean difference of 0.22 mm (*P* < 0.0001). The correlation between the ACD values from the two devices was good (*r* = 0.80) (Figure 1).  Figure 2 shows the Bland–Altman plots of these data.

**Figure 3:**
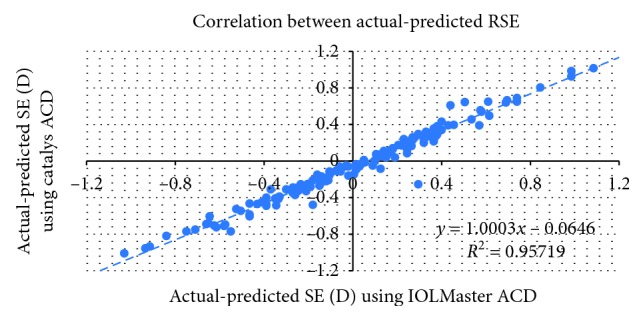
Correlation between prediction errors of both devices.

**Figure 4:**
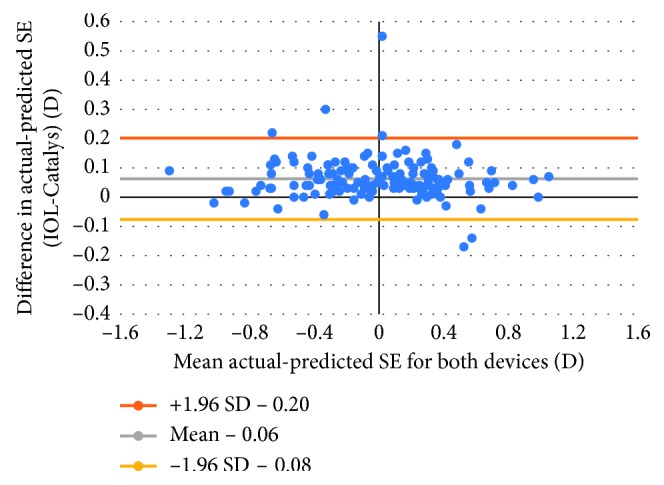
Bland–Altman plot of prediction errors for both devices.

Table 2 shows the mean, standard deviation, minimum and maximum absolute prediction errors for each device and their difference. There was no statistically significant difference between the MAE derived from the IOLMaster, 0.37 D ± 0.34 (SD) and the Catalys, 0.37 D ± 0.35 (SD) (*P*=0.50). The correlation between the postoperative and predicted RSE of the implanted IOL power was excellent (*r* = 0.96) (Figure 3).  Figure 4 shows the Bland–Altman plots of these data.

**Figure 5:**
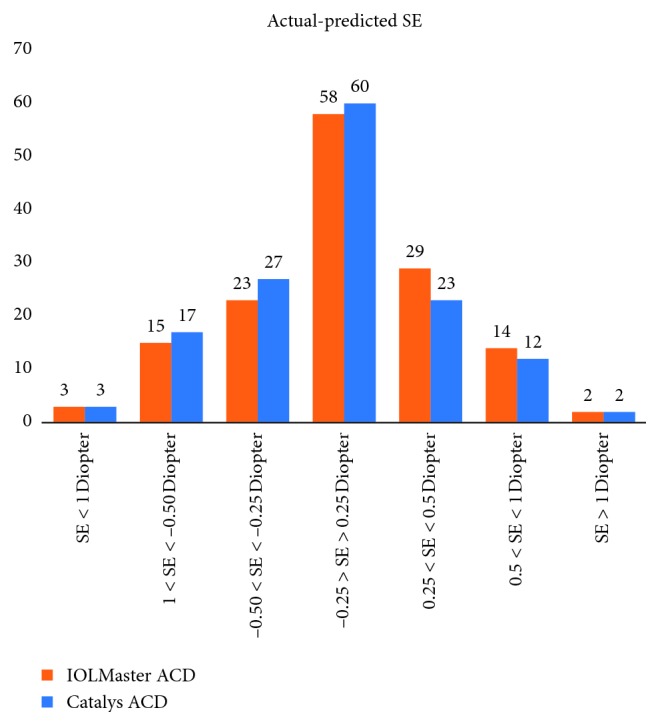
Graph of prediction errors for both devices.

The percentage of eyes with a measured RSE within 0.25, 0.50, 0.75, and 1.0 D of predicted were equal in both groups. In the Catalys group, 58 eyes (40%) were within 0.25 D, 110 (76%) within 0.50 D, 130 (90%) within 0.75 D, and 136 (94%) within 1.0 D. In the IOLMaster group, 57 eyes (40%) were within 0.25 D, 109 (76%) within 0.50 D, 129 (90%) within 0.75 D, and 136 (94%) within 1.0 D. In each group, 5 eyes (3.4%) had a measured RSE greater than 1.0 D of predicted values.  Figure 5 shows a graphical representation of these data. Fisher's exact test was conducted and resulted in no significant difference in the ratio of RSE less than 0.50 D of predicted between the Catalys and IOLMaster.

## 4. Discussion

Achieving ideal refractive outcomes in cataract surgery relies on the ability to accurately predict ELP and postoperative ACD. Precise measurements of certain preoperative biometric measurements such as ACD, in addition to the choice of IOL power formula, are crucial in accurately estimating ELP. This study compares preoperative ACD measurements between two devices and evaluates their refractive predictability.

To our knowledge, this study is the first to compare the accuracy of intraocular lens power calculation and ACD measurements taken with the Catalys OCT system. Many previous studies have compared ACD measurements taken by other devices, most commonly the Lenstar-LS900 (Haag-Streit AG, Koeniz, Switzerland) [11, 17–19].  Table 3 compares our ACD measurements with other studies using different devices. Introduced in 2009, the Lenstar was the first device to provide biometric measurements using optical low coherence reflectometry (OLCR) that correlated well with those of the IOLMaster [18, 19]. Reitblat et al. [10] compared the IOLMaster 500 with the Lenstar and reported the Lenstar to measure a deeper ACD with a mean difference of 0.07 (*P* < 0.001). Rabsilber et al. [17] also found ACD measurements taken by the Lenstar to be larger compared to those taken by the IOLMaster 500. Goebels et al. [20] compared ocular biometry taken by the IOLMaster 500, Lenstar, and OA-2000, an OLCR based device (Tomey, Nagoya, Japan), and concluded that biometric measurements correlated well, with the OA-2000 yielding statistically significant larger mean ACD (0.2 mm) compared to IOLMaster. These studies parallel our results and suggest that the IOLMaster tends to measure a shallower ACD compared to other biometric devices.

Kaswin et al. [2] compared biometry taken by the IOLMaster 500 with the AL-Scan (Nidek Co., Ltd.), a newer PCI-based optical biometer, and also found good agreement (*r* = 0.701) between ACD measurements, with mean ACD values measuring 0.13 ± 0.04 mm larger in the AL-Scan group compared to those of the IOLMaster group. Despite the variability in ACD measurements, IOL power calculations using the Haigis formula were highly comparable; the mean IOL power difference was 0.50 D or less between the 2 devices in 94% of cases. Our study found a larger difference in ACD measurement between the Catalys and IOLMaster (0.22 mm). Though their study found a slightly lower MAE in the AL-Scan group compared to IOLMaster, our study found no significant difference in the MAE derived from the Catalys and IOLMaster.

While the IOLMaster and Catalys ACD values correlated well, we found that the Catalys measured an ACD that was significantly deeper than the IOLMaster. This could be attributed to the difference in the technique—the IOLMaster uses lateral slit illumination whereas the Catalys uses OCT to measure ACD. Of note, patient's head posture also differs when obtaining measurements, possibly contributing to a change in the ocular lens position and ACD. In our study, patients were positioned supine during Catalys measurement and seated upright with the IOLMaster. No study has confirmed the effect of gravity manifested by a supine head position in ACD measurement; one study, however, found that a prone head position was associated with a small decrease in ACD measurement (0.04–0.12 mm) compared to an upright head position in patients measured by the Lenstar [21]. Based on these results, gravity alone would not account for the entire difference in ACD measurement between the two devices in our study.

Although values in ACD measurements differed, our study did not reveal a clinical difference in absolute prediction error using the biometry from either system at 1-month postoperative follow-up. One possible reason is that the difference in ACD measurement may only have a relatively minor effect in the Barrett Universal II formula algorithm compared to other biometric variables, which remained unchanged. In addition, because the mean absolute errors achieved by both devices in this study are minimal, the difference in ACD alone may be insufficient in achieving a higher prediction accuracy through current methodology and prediction algorithms.

Limitations in this study include its retrospective design and limited sample size. A large prospective study would be ideal to further evaluate the accuracy of IOL power calculation. In addition, eyes were not controlled for pupillary dilation, which may affect anterior chamber depth measurement. Previous papers have shown that pupil dilation results in increased ACD measurement calculated by the IOLMaster [22–24]. More studies are needed to compare the effect of pupillary dilation using the IOLMaster.

Furthermore, since Catalys biometry is obtained minutes prior to the operation, the clinical application of these measurements is uncertain as surgeons may not have the time to select the suggested IOL power if no formula is automatically integrated within the Catalys system. The ACD measured by the Catalys is calculated moments before cataract surgery, long after the IOL power had already been selected. Regardless, future studies utilizing other biometric values provided by the Catalys and IOL power formulas would be worthwhile. A software update with integration of IOL calculation in the femtosecond laser may render this limitation obsolete in the future.

In conclusion, the difference in ACD measurements generated by the Catalys and IOLMaster were statistically but not clinically significant given that the ACD calculated by the Catalys is measured only minutes before the operation, after the IOL power had long been determined. The IOLMaster has been considered the gold standard device for biometry and measurements can be taken preoperatively. The Catalys is an integrated femtosecond laser phacoemulsification system combined with optical technology that provides biometric measurements minutes prior to surgical intervention. Further studies are needed to compare these devices evaluating other biometric values and IOL power formulas.

## Figures and Tables

**Table 1:** ACD mean, standard deviation, and minimum and maximum.

	IOLMaster *n* = 144	Catalys *n* = 144	Difference
Average	3.14 ± 0.41	3.37 ± 0.40	−0.22 ± 0.17 *P* < 0.0001
Minimum	2.18	2.40	
Maximum	4.14	4.20	

**Table 2:** Prediction error mean, standard deviation, and minimum and maximum.

	IOLMaster *n* = 144	Catalys *n* = 144	Difference
Average	0.37 ± 0.34	0.37 ± 0.35	−0.004 ± 0.08 *P*=0.50
Minimum	0	0	
Maximum	2.85	2.92	

**Table 3:** Comparison with other studies.

	Device	Mean ACD ± SD (mm)	*P* value
Present study *N* = 144	IOLMaster 500	3.14 ± 0.41	*P* < 0.0001
	Catalys	3.37 ± 0.40	

Reitblat et al. [10] *N* = 73	IOLMaster 500	3.35 ± 034	*P* < 0.0001
	Lenstar	3.42 ± 0.34	

Goebels et al. [20] *N* = 138	IOLMaster 500	3.0 ± 0.45	*P* < 0.0001
	Lenstar	3.09 ± 0.47	
	OA-2000	3.71 ± 0.51	

Kaswin et al. [2] *N* = 50	IOLMaster 500	3.12 ± 0.38	—
	AL-Scan	3.17 ± 0.41	

## Data Availability

The data used to support the findings of this study are restricted in order to protect patient privacy. Data are available from the corresponding author for researchers who meet the criteria for access to confidential data.
